# Effect of Nanodiamond Concentration and the Current Density of the Electrolyte on the Texture and Mechanical Properties of Ni/Nanodiamond Composite Coatings Produced by Electrodeposition

**DOI:** 10.3390/ma12071105

**Published:** 2019-04-03

**Authors:** Meihua Liu, Hongnan Liu, Dongai Wang, Bing Liu, Yan Shi, Feihui Li, Yunlan Gong, Linan Li, Lianjin Li, Wengang Zhang

**Affiliations:** 1 Department of Mechanics, Tianjin University of Commerce, Tianjin 300134, China; lmhua2007bd@126.com (M.L.); lhnan@tjcu.edu.cn (H.L.); liubing19851126@163.com (B.L.); shyan@tjcu.edu.cn (Y.S.); lilianjin@163.com (L.L.); 2 Science College, Tianjin University of Commerce, Tianjin 300134, China; tjlifeihui@tjcu.edu.cn (F.L.); daniewang@hotmail.com (Y.G.); 3 Department of Mechanics, Tianjin University, Tianjin 300072, China; lali@tju.edu.cn; 4 Tianjin Chanyu Superhard Sci-Tech Co., Ltd., Tianjin 300384, China; cnchanyu@163.com

**Keywords:** nanodiamond, electroplated composite coating, texture, friction property

## Abstract

An Ni/nanodiamond composite coating was deposited on carbon steel in a traditional Watt’s solution without additives via direct current (DC) electroplating. The effects of the nanodiamond concentration and current density in the plating solution on the morphology, grain size, and texture of the Ni/nanodiamond composite coating were observed using X-ray diffraction (XRD) and scanning electron microscopy (SEM). The distribution of the nanodiamond particles in the composite coating was investigated by Raman spectra and SEM. The mechanical properties of the composite coating, such as its elastic modulus and hardness, were examined using a Nano Indenter XP nanometer mechanical test system. The coefficient of friction was tested using a Universal Micro-Tribotester. The results demonstrated that the preferential orientation of the Ni/nanodiamond composite coating varied from the (111) crystal orientation of the pure nickel coating to the (200) crystal orientation. When the nanodiamond concentration in the plating solution was 8.0 g/L and the current density was 3.0 A/dm^2^, the hardness of the composite coating reached the maximum value of 5.302 GPa and the friction factor was maintained at around 0.1. The average grain size of the composite coating was reduced to 20.4 nm.

## 1. Introduction

Particle-reinforced metal matrix composites have many advantages, such as a low cost, corresponding high hardness, good wear resistance and corrosion resistance compared to pure metals or alloys [1,2,3,4,5,6,7,8,9,10,11,12,13,14,15,16,17,18,19]. Composite electroplating has been identified as a technologically feasible and economically superior technique for the preparation of such composites since its temperature of operation is precisely controlled to be near the ambient value, it uses normal pressure conditions, and it has a low cost, a rapid deposition rate, is simple to scale-up, and all the necessary equipment is easily maintained. Over the past three decades, the successful co-deposition of micro- and nano-sized inorganic particles such as SiC [2,3,4,5,6,7,8], WC [9], Al_2_O_3_ [10,11], and diamond [12,13,14,15,16,17,18,19,20] with metals or alloys using the conventional electroplating technique has been reported.

Researchers are paying more and more attention to the Ni matrix reinforced with nanoparticles, especially nanodiamond particles [12,13,14,15,16,17,18,19,20]. Unique mechanical and tribological properties, including high hardness and a low friction coefficient of the ultra-fine nanodiamond powders, have attracted researchers to conduct further investigation into their utilization as coatings and as composite coatings. A considerable amount of research has been focused on the impact of the electrodeposition parameters on the electrolytic co-deposition of nanodiamond particles with Ni and the properties of the composite coating. These parameters included the electrolysis conditions (composition of electrolytic bath, presence of additives, pH value) [17,18], the current conditions (such as the imposed current and current density values), and the properties of the reinforcing particles, such as particle size, surface properties, concentration, and type of dispersion in the bath.

Compared to the ordinary composite coating, the nanocomposite coating has improved hardness, abrasion resistance, and friction reduction. At present, the study of nanocomposite coating is mainly limited to the addition of various nanoparticles and the utilization of various plating solution components or substrates. Consequently, the corresponding study scope, depth, and breadth are insufficient.

In this study, the Ni/nanodiamond composite coatings were electroplated using the direct current electrodeposition method using a Watts-type bath without any additives, containing nanodiamond particles only. The microstructure, texture, and mechanical properties of the resulting composites were investigated. For comparison, a pure nickel coating and an Ni/nanodiamond composite coating were also produced using the conventional electroplating technique. The affecting law of deposited current density and the nanodiamond concentration in the plating solution on the mechanical properties of the Ni/nanodiamond composite coating deposited on Q235A low-carbon steel was studied. The crystal structure and residual stress of the composite coating were analyzed, the morphology and the distribution of the nanodiamond particles were observed, and the hardness and elastic modulus were tested. This laid a foundation for the corresponding establishment of the relationship between the mechanical properties and the electroplating technological parameters of composite coatings, and also provided technical support for the preparation of high-strength Ni/nanodiamond composite coatings.

## 2. Experimental Procedure

### 2.1. Substrate Pretreatment

**Figure 1:**
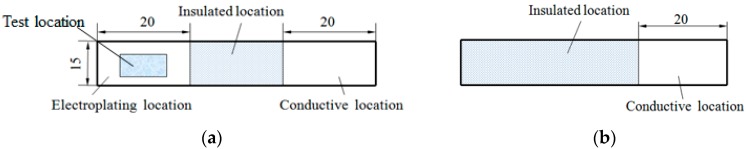
Schematic diagram of the matrix size of the substrate and the sealing, plating, and conductive locations: (**a**) front view and (**b**) back-face view.

Q235A mild steel (wt.%: 0.14–0.22 C, 0.30–0.65 Mn, ≤0.50 S, ≤0.45 P, ≤0.30 Si) was utilized as the substrate with dimensions of 60 mm × 15 mm × 2 mm, as presented in Figure 1. For each substrate, the coated area was set as 20 mm × 15 mm while the remaining part was sealed by an insulating material. The 10 mm × 5 mm test area was confined to the area represented by the shadowed part of Figure 1a, in the middle of the coating layer. The process flow was as follows: mechanical polishing–distilled water rinsing–chemical de-oiling–distilled water rinsing–electrochemical de-oiling–distilled water rinsing–electroplating–distilled water rinsing–drying.

Firstly, the Q235A matrix was ground with the No. 60 and 80 corundum grinding wheels and subsequently polished with the No. 300 and 800 soft grinding stones until the corresponding surface had no significant scratches. The chemical degreasing, electrochemical degreasing, and acidification were as presented in Table 1.

The chemical degreasing was first conducted in the beaker, and consequently, the electrochemical degreasing was executed with the Q235A as the cathode and the platinum mesh as the anode. The electrolyte was prepared from analytical grade chemicals and distilled water. All experimental chemicals were provided by the Tianjin Guangfu Fine Chemicals Research Center, Tianjin, China. To enhance the binding force between the coating and the matrix, the Q235A matrix was rinsed with distilled water and then immersed in a 3% HCl solution at room temperature for 10 s prior to plating. The chemical degreasing was conducted using DC regulated power supply, HX1731LL2A, made in Ningbo Hexing Electronics Co. Ltd., Ningbo, China.

### 2.2. Composite Solution and Process Conditions

The electrodeposition of Ni was performed using a Watts baths containing NiSO_4_ 6H_2_O 240.0 g/L (calculated as Ni ion), NiCl_2_ 6H_2_O 20.0 g/L (calculated as Ni ion), H_3_BO_3_ 20.0 g/L, and sodium dodecyl sulfate 0.5 g/L. A range of nanodiamond concentrations was co-deposited with Ni as follows: 0, 0.8, 4, 8, and 16 g/L. The nanodiamond particles used in the study were synthesized by the detonation synthesis method with an average particle size of 5 nm.

The composite electroplating process conditions and device were as follows [21]: the cathode current density ranged from 2 to 5 A/dm^2^, the PH value was 3.5, the temperature was 45 °C, and the time period was 70 min; a DF-101S magnetic stirrer, with a speed of 100 r/min, was used, with a platinum mesh as the anode, with dimensions of 80 mm × 50 mm. The Ni/nanodiamond composite coating thickness varied but was around 20 μm.

**Figure 2:**
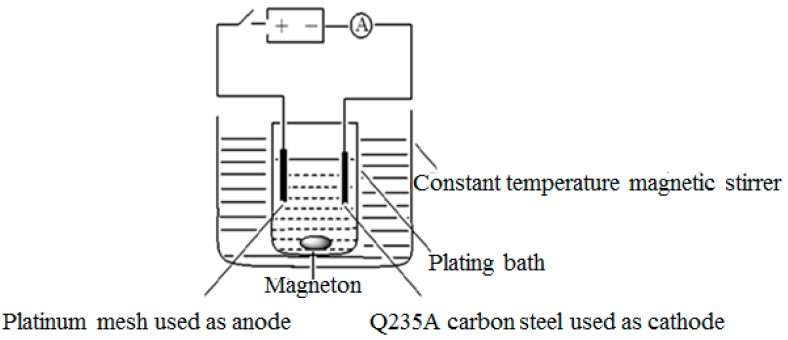
Schematic diagram of the Ni/nanodiamond composite plating device.

The electroplating device is presented in Figure 2.

The nanodiamond particles used in the present study were synthesized by the detonation synthesis method with an average particle size of the diamond of 5 nm. The main components were carbon 88%~93%, water ≤ 3%, nitrogen ≤ 2.5%, and oxygen ≤ 10% [22]. The contents of the impure elements (gas elements excluded) in the sample were determined by atomic emission spectroscopy, prepared by Tianjin Chanyu Superhard Sci-Tech Co., Ltd., Tianjin, China, and are listed in Table 2.

### 2.3. Test Analysis

The crystal structure of the Ni/nanodiamond composite coating was analyzed using a D/MAX-2500 X-ray diffractometer (Rigaku, Tokyo, Japan), and the parameters were as follows: copper target, a tube voltage of 40 kV, and a scanning rate of 8 °/min. The (400) crystal orientation was selected to test the residual stress, with an inclination of 0° to 45°.

The surface morphology of the Ni/nanodiamond composite coating was observed using a Hitachi X650 scanning electron microscope (Tokyo, Japan), and the grain distribution was observed using a voltage of 30 kV. The voltage was magnified by between 5000 and 10,000 times. Simultaneously, the composition was analyzed with the energy dispersive spectrometer (EDS) of the scanning electron microscope. The Nano Indenter XP nanometer mechanical test system (American MTS Company, Monroe, NC, USA) was utilized to measure the hardness with a Berkovich diamond as the indenter; the loading or unloading rate was 40 nm/s and the pressed depth was 2500 nm for 10 s. The coefficient of friction was tested using a UMT-2 (Center of tribology, Campbell, CA, USA), at a sliding velocity of 120 r/min and an initial load at the point of contact of 5 N. Steel balls (bearing steel GCr15) with a diameter of 8 mm were used. The tests were performed in air without any lubricants for 1 min. The wear was evaluated using scanning electron microscopy (SEM), LEO 1530 VP. Raman spectra were taken on a RM2000 micro confocal Raman spectrometer produced by RENISHAW (Wharton Anderch, UK). The excitation wavelength was 514.5 nm, and the scanning range was from 1000 to 2000 cm^−1^ with a step of 1 cm^−1^.

In order to describe the structure and quantitatively acquire the preferential orientation of the deposits, the relative texture coefficient was calculated as follows [23,24]:

(1) $${\mathrm{RTC}}_{\mathrm{hkl}}=\frac{I_{\left(\mathrm{hkl}\right)}/I_{\left(\mathrm{hk}l\right)}^{0}}{\sum_{i=1}^{n}I_{\left(\mathrm{hkl}\right)}/I_{\left(\mathrm{hkl}\right)}^{0}}\times 100\%$$

where *I*_(hkl)_ is the diffracted intensity of the (hkl) lines determined by the diffractogram of the deposits and *I*_(hkl)_^0^ is the corresponding intensity of the standard nickel sample randomly orientated from JCPDS 04-0850. The parameter *n* is the number of diffraction peaks. When the value of RTC_hkl_ exceeded the average value, the preferential orientation of the (hkl) crystal face occurred.

The grain size of the coating was calculated according to the Scherrer equation [25]:

(2) $$d=\frac{0.9\lambda }{B\cos\theta }$$

where λ is the wavelength of the X-ray, (nm) and θ is the Bragg diffraction angle, (°). *B* is the full width at the half maximum height (FWHM) of the peak.

## 3. Results and Discussion

### 3.1. Effects of Nanodiamond Concentration on the Cathodic Polarization Curves

**Figure 3:**
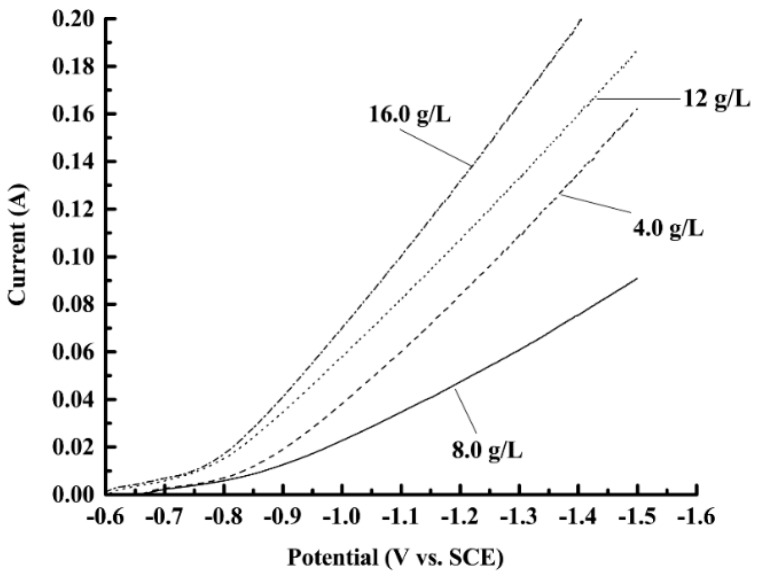
Cathodic polarization curves tested in solutions containing different concentrations of nanodiamond powder.

Figure 3 shows the cathodic polarization curves measured in the bath with different nanodiamond concentrations. It can be seen that the current is low when the potential is in the range −0.6~−0.8 V, which indicated that the substantial reduction of Ni^2+^ had not yet started, and therefore the current was small. When the potential was lower than −0.8 V, the reduction of Ni^2+^ started to accelerate and the current increased significantly. Comparing the over-potential of the different curves under the same current, it can be found that the over-potential first increased then decreased with the increase of nanodiamond concentration; the highest over-potential was obtained when the nanodiamond concentration in the plating solution was 8 g/L. The results may be due to the fact that the adsorption and deposition of nanodiamond particles on the surface of the electrode has a certain hindering effect on the discharge of metal ions, which is favorable for the deposition of nanodiamond particles in the coating. However, when the nanodiamond content in the bath is too high, the hindering effect becomes weakened, and the reduction rate of Ni^2+^ is accelerated, which leads to a decrease of the nanodiamond quantity in the composite coating.

### 3.2. Effect of the Nanodiamond Concentration in the Plating Solution on the Morphology and Phase Structure of Composite Coating

#### 3.2.1. Surface Morphology

**Figure 4:**
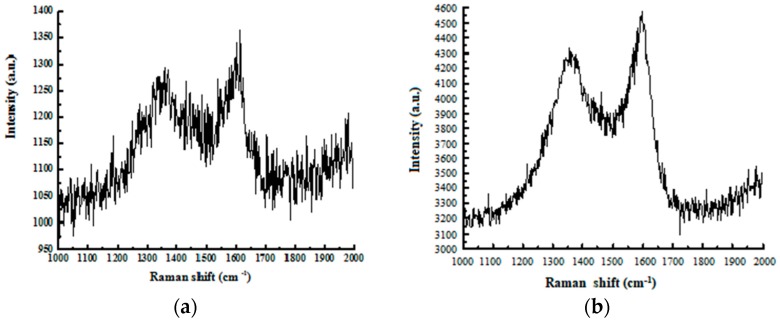

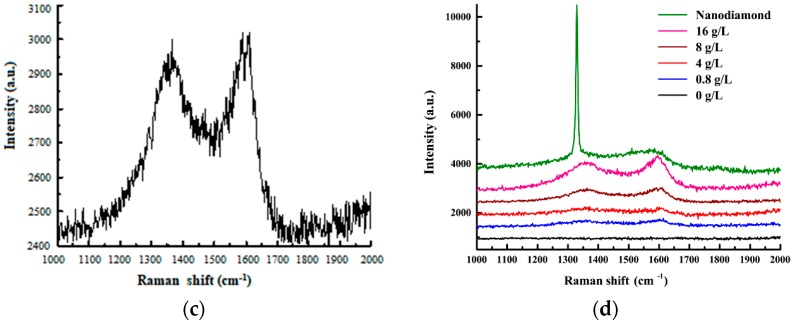
The Raman spectra of the composite coating deposited at various concentrations of nanodiamond particles in the plating solution from 0 to 16.0 g/L: (**a**) 4 g/L, (**b**) 8 g/L, (**c**) 16 g/L, and (**d**) a multiple comparison study.

Figure 4 shows the Raman spectra of the composite coatings accompanied by the spectrum of the pure nickel film as a control group. Two evidently widened peaks could be observed near 1350 and 1600 cm^−1^ for all the samples, except for the pure nickel film. By increasing the concentration of nanodiamond particles in the plating solution, the intensity of both peaks also increased. These two signals verified the sp^3^ and sp^2^ hybridization of C–C bonding in the composite coating, which provided strong evidence of the existence of both diamond and graphite. However, all peaks were broadened and suffered from a low signal-to-noise ratio, except for the sample prepared from the plating solution with a 16 g/L nanodiamond concentration.

The intensity, position, and full width at half maximum (FWHM) of the Raman peak are strongly related to the intrinsic properties of the sample, such as the degree of crystallinity, concentration of defects, and inner stress. According to the literature [26], a single crystal diamond has a sharp Raman peak at 1332 cm^−1^, attributed to the sp^3^ hybridization of C–C bonding in its regular tetrahedral crystal field. Graphite, however, presents a strong signal at 1576 cm^−1^, which is related to the sp^2^ hybridization of the C–C bonding interlayer. In our experiments, the broadened and weakened Raman peaks can be understood from two aspects. On one hand, the nanodiamond particles were prepared by a negative oxygen balanced explosive detonation method. The obtained nanodiamond had high surface energy, and the perfect diamond crystal structure could not be fully retained. On the other hand, the high concentration of defects in the coatings may cause the interaction between nanodiamond particles and surrounding atoms (e.g., the formation of C–Ni bonding). Catledge and Vohra claimed [27] that inner stress would cause a shift of the Raman peaks from 1332 to 1350 cm^−1^, which is agrees very well with our observation [28].

**Figure 5:**
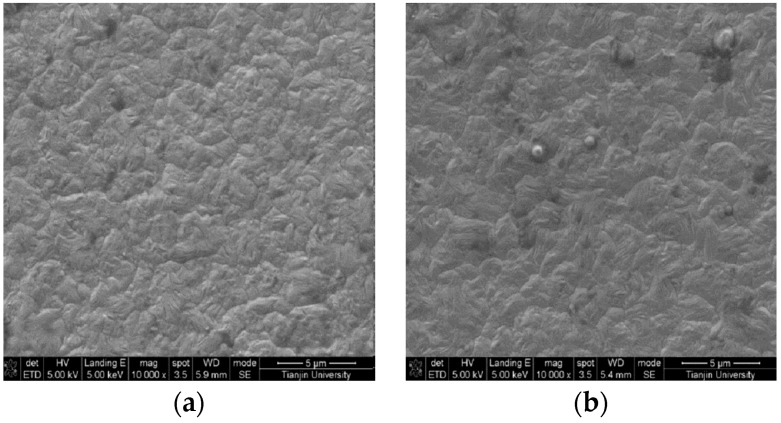

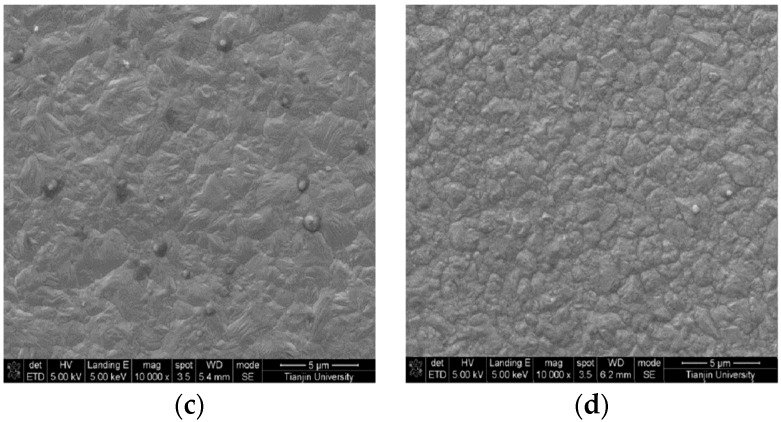
Scanning electron microscopy (SEM) images of the composite coating plated at various concentrations of nanodiamond particles in the plating solution from 0.8 to 16.0 g/L: (**a**) 0.8 g/L, (**b**) 4.0 g/L, (**c**) 8.0 g/L, and (**d**) 16.0 g/L.

Figure 5 presents the surface morphology of the Ni/nanodiamond composite coating at the current density of 3.0 A/dm^2^ and with various nanodiamond concentrations in the plating solution. It could be observed that the surface morphology of the composite coating was relatively dense and the grain was compact when the nanodiamond particles were contained in the plating solution. As the nanodiamond concentration in the plating solution increased, the content of nanodiamond particles in the composite coating also increased. The smaller the surface grains are, the more uniform the distribution is. This is because nanodiamond particles dispersed in matrix metals can promote nucleation, effectively preventing the growth of grains and refining the surface. When the nanodiamond particles were completely embedded into the nickel matrix, the composite coating would sustain the dispersion strengthening effect. Certain particle sizes were higher than micron level, which indicated that the agglomeration phenomenon occurred for a portion of the nanodiamond particles in the plating solution.

#### 3.2.2. Phase Structure

**Figure 6:**
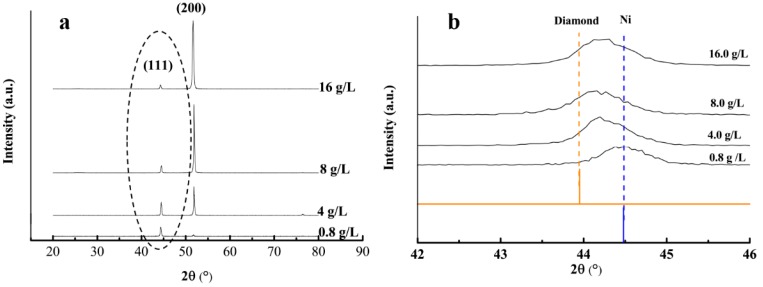
(**a**) Full X-ray diffraction (XRD) spectrum of the composite coating prepared with various nanodiamond concentrations in the plating solution and (**b**) the partial magnification of the XRD spectra marked within a dashed ellipse in (**a**).

Figure 6 presents the X-ray diffraction (XRD) patterns of the plated deposits and the texture coefficients of the (200) crystal face at the current density of 3.0 A/dm^2^ and with various nanodiamond concentrations in the plating solution. According to Figure 6a, no new phase was formed following the addition of nanodiamond particles, whereas the diffraction intensity of the various crystal faces of the composite coating was significantly changed. The diffraction peak intensity of the (200) crystal orientation in the composite coating gradually increased as the nanodiamond concentration increased, indicating that the addition of nanodiamond particles led to the preferential orientation of the composite coating along the (200) crystal orientation.

Figure 6b shows the partial magnification of the XRD spectra marked within a dashed ellipse in Figure 6a, which demonstrates the existence of nanodiamond particles. It can be clearly observed that when the concentration of the nanodiamond particles in the plating solution varies from 0.8 g/L to 16.0 g/L, all the (111) peaks of the composite coatings deviate from the (111) peak of standard nickel. When the concentration of the nanodiamond particles in the plating solution was 8.0 g/L, the peak position of the (111) reflection was closer to the peak of standard diamond compared to the other three coatings, indicating that this concentration results in the highest nanodiamond-to-nickel ratio in the coating.

**Figure 7:**
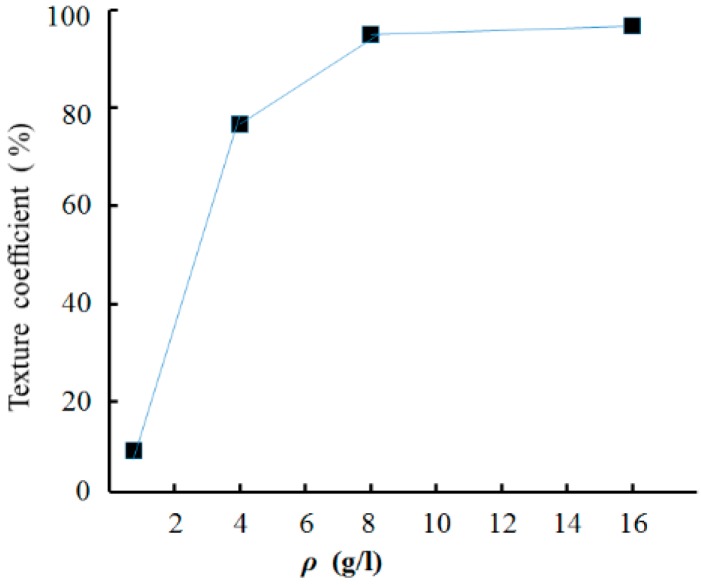
Texture coefficients of (200) crystal orientation with various nanodiamond concentrations in the plating solution.

Figure 7 shows that the texture coefficient of the (200) crystal orientation reached 95.5% when the nanodiamond concentration in the plating solution was 16.0 g/L.

### 3.3. Effect of Current Density on the Surface Morphology and Phase Structure of the Composite Coating

#### 3.3.1. Surface Morphology

**Figure 8:**
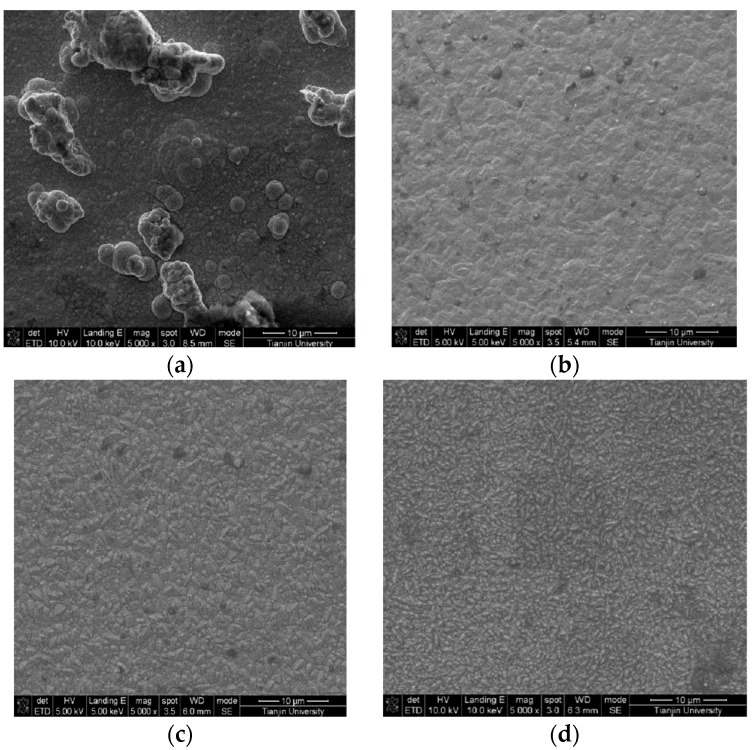
SEM images of the composite coating prepared at various current densities: (**a**) 2.0 A/dm^2^; (**b**) 3.0 A/dm^2^; (**c**) 4.0 A/dm^2^ and (**d**) 5.0 A/dm^2^.

Figure 8 presents the surface morphology of the Ni/nanodiamond composite coating with a nanodiamond concentration of 8.0 g/L in the plating solution at various current densities from 2.0 to 5.0 A/dm^2^. It can be observed that the higher the current density, the lower the resulting grain size on the surface of the composite coating. When the current density was 2.0 A/dm^2^, the grains in the composite coating formed a cauliflower shape. Additionally, as the current density gradually increased, the grain shape changed into a prominent pyramid polyhedron shape and finally into an elongated columnar shape. The results demonstrated that the change in the current density had a significant effect on the grain shape in the composite coating, which changed from the cauliflower shape to the columnar structure.

#### 3.3.2. Phase Structure

**Figure 9:**
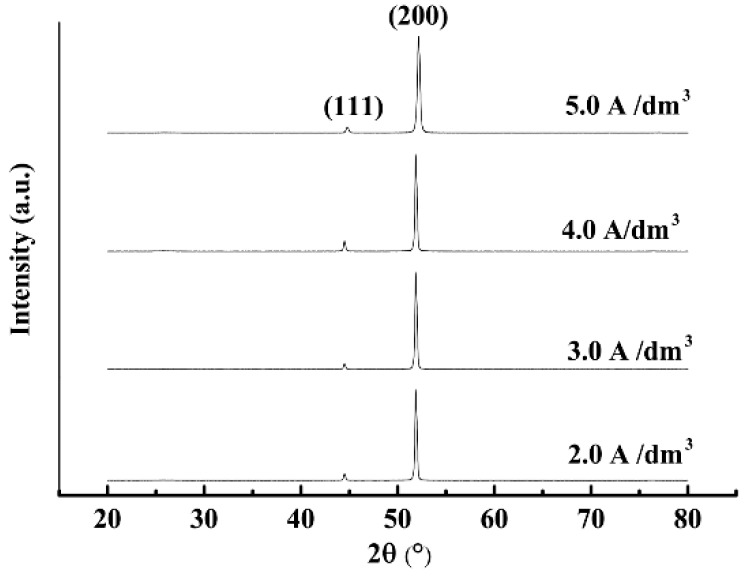
XRD pattern of the composite coating at various current densities.

Figure 9 presents the XRD pattern of the Ni/nanodiamond composite coating prepared at various current densities. When the plating current density increased, the intensity of the (111) and (200) peaks of the composite coating displayed slight changes. The intensity of the composite coating crystal orientation showed a similar trend as a function of the current densities. From the XRD patterns, an apparent change in the crystallographic structure was observed according to the intensity of the (200) and (111) diffraction peaks, and the (200) preferential orientation developed accordingly. This phenomenon demonstrated that the crystallographic orientation was not affected by the current density used in the composite electroplating of the Ni/nanodiamond particles.

**Figure 10:**
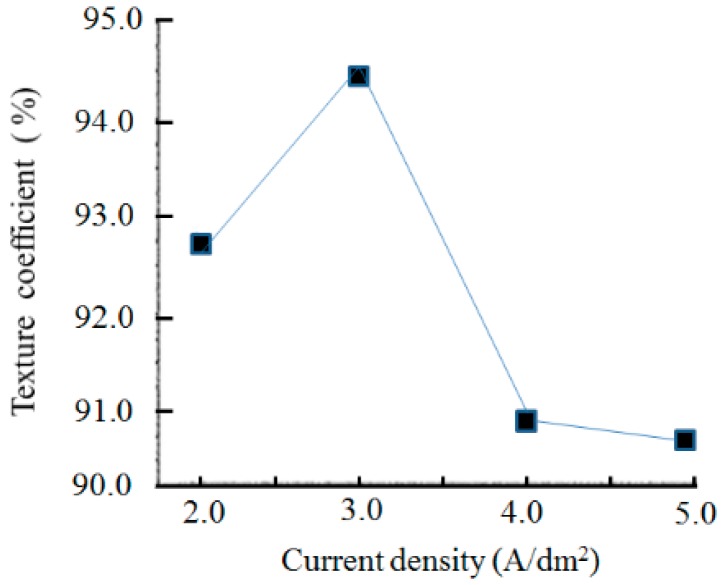
Texture coefficients of (200) crystal orientation at various current densities.

Figure 10 presents the effect of current densities on texture coefficients of the (200) crystal orientation with a nanodiamond concentration of 8.0 g/L in the plating solution. The texture coefficient of the (200) crystal orientation first increased and then decreased with the increase of the current density; however, the differences were slight and the percentages of the texture coefficient exceeded 90%. When the current density was 3.0 A/dm^2^, the texture coefficient of the (200) crystal orientation reached the maximum value of 94.47%, and no new phase in the Ni/nanodiamond composite coating was apparent. The grain size was calculated from the Scherrer equation [25]. The result demonstrated that grain refinement was observed as the current density increased. Moreover, the grain size of the composite coating reached a minimum value of 20.4 nm.

### 3.4. Effect of Nanodiamond Concentration in the Plating Solution and Current Density on the Hardness and Wear Resistance of the Composite Coating

**Figure 11:**
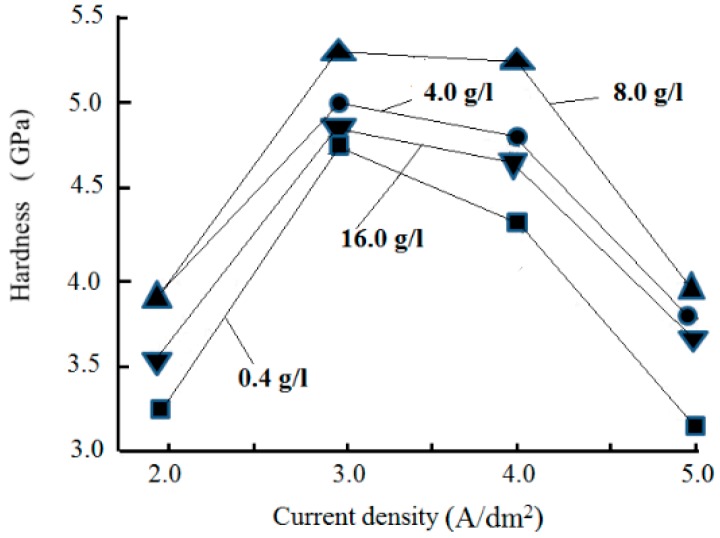
Effect of nanodiamond concentration in the plating solution on the hardness of the composite coating.

Figure 11 presents the effect of the nanodiamond concentration in the plating solution on the composite coating hardness at various current densities. Each point is the average value of the results obtained from five randomly chosen spots.

The composition of the coating was analyzed qualitatively using the energy dispersive spectrometer (EDS) of the scanning electron microscope. The results showed that the carbon content of the composite coating increased as the nanodiamond concentration in the plating solution increased. When the nanodiamond concentration in the plating solution was 8.0 g/L and the current density was at 3.0 A/dm^2^, the carbon content of the composite coating reached a maximum, and the hardness of the composite coating reached a maximum of 5.302 GPa. When the nanodiamond concentration exceeded 8.0 g/L in the plating solution, the carbon content of the composite coating began to decrease. Upon analysis of the test results, we found that the data tested on the same measured sample have different degrees of dispersivity. In particular, when the nanodiamond content in the plating solution is 16 g/L, the results are more dispersed. We infer that this may be caused by the agglomeration of nanodiamond particles in the plating solution.

**Figure 12:**
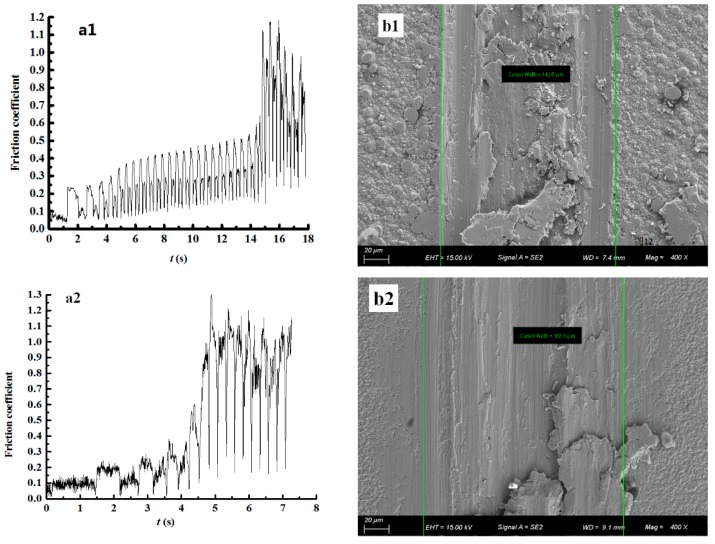

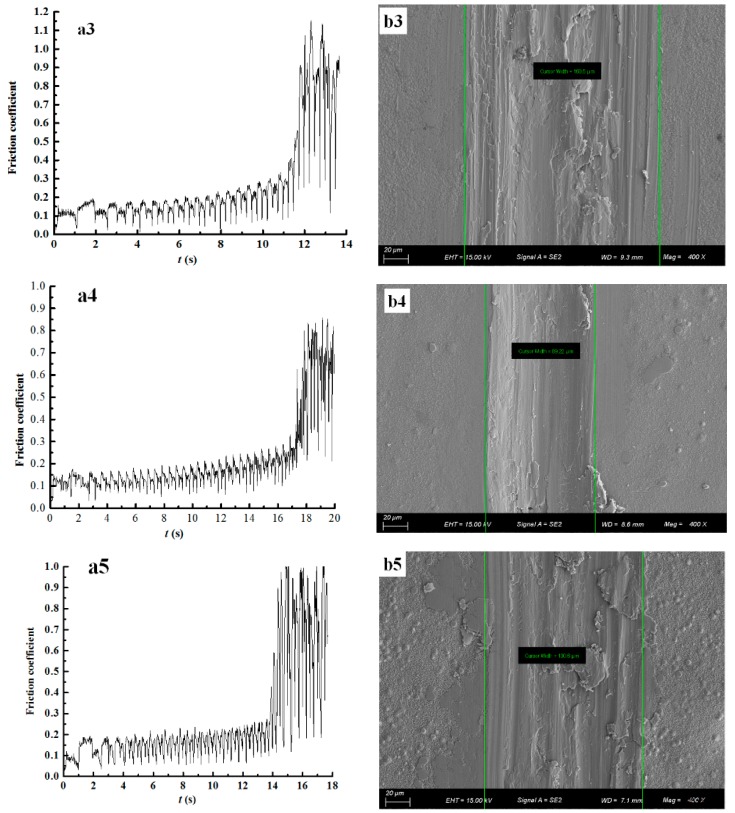
The abrasion resistance of coatings. (**a**) The friction factor and (**b**) corresponding SEM images of wear scar width. The numbers 1 to 5 represent that the concentration of the nanodiamond particles in the plating solution is 0, 0.8, 4.0, 8.0, and 16.0 g/L, respectively.

Next, the abrasion resistance of the composite coating was further evaluated using the friction factor and visualization of the wear scar width. Figure 12 presents the results of coatings when the concentration of the nanodiamond particles in the plating solution is 0, 0.8, 4.0, 8.0, and 16.0 g/L, respectively.

According to Figure 12a, in the first ~6 s, the friction factor of the Ni coating is around 0.3 (Figure 12a1)—the friction factors of all the composite coatings are in the range of 0.1 to 0.15 (Figure 12a2–a5)—while the friction factor of the coating prepared with an 8.0 g/L nanodiamond concentration remains around 0.1 (Figure 12a4). As the friction time increases, the heat generated will cause surface warming, which may lead to adhesion abrasion. By combining the SEM results shown in Figure 12b, the coating prepared with a nanodiamond concentration of 8.0 g/L is more wear-resistant, which is consistent with the conclusion obtained from Figure 12b4.

A contrastive analysis of properties was made between the Ni coating and Ni/nanodiamond composite coatings by Petrov et al [13]. The conclusion demonstrated that the incorporation of nanodiamond particles significantly affects the tribological properties of the corresponding composites. In our experiments, by combining with bearing steel GCr15 as friction pairs, the pure nickel film presented a greater friction factor than the Ni/nanodiamond composite coating. In the 15 s measurement, the friction factor varied around 0.3 (Figure 12a1) and the nickel surface after testing showed severe adhesive wear (Figure 12b1). This mainly stems from the poor bearing capacity of the pure nickel film with low hardness, which will lead to coating softness and adhesion peeling caused by surface warming. The friction factors of all the nanodiamond composite coatings were in the range of approximately 0.1–0.15, smaller than that of pure nickel film (Figure 12a2–a5). The composite coating prepared with 8.0 g/L nanodiamond content in the plating bath had the lowest friction factor (0.12), as shown in Figure 12a4. Even though evident adhesion and ploughing can be observed, large-scale exfoliation did not occur. In particular, when the concentration of nanodiamond particles in the plating solution was 8.0 g/L, only mild adhesion and friction was observed (Figure 12b4).

The main problem of composite materials, in general, is the reinforcement matrix interface. Therefore, in-depth research on bonding strength between the composite coating and base is very important. Molina et al. developed a method for the electrochemical deposition of nickel on Al composites [29,30]. This method serves to modify surface materials or even composite materials, resulting in coatings that are highly resistant to wear and have a good adherence; further research on this will be carried out.

Evidently, the introduction of nanodiamond particles can effectively improve the wear-resistance property of the composite coating, and the origin of this phenomenon is attributed to, firstly, a dispersion/dislocation strengthening effect and secondly, a grain refinement strengthening effect of the nanoparticles. On one hand, as the nanodiamond particles are randomly and uniformly distributed in the coating, severe lattice distortion occurs and thus increases the defect concentration. In the substrate, the movement and deformation of nickel are hindered, which will result in a stronger composite coating and an enhanced wear-resistance property. On the other hand, the integration of nanodiamond particles into the composite coating is a non-spontaneous nucleation process instead of in-situ growth during chemical plating, which consequently leads to grain refinement. As dislocations move inside the polycrystal, the sliding resistance is enhanced because of the different crystal orientations near the grain boundary and the accumulated impurities nearby. Accordingly, this causes the slip band on one side to have difficulty sliding into other crystal grains. Furthermore, this behavior would be even more difficult to achieve considering the fact that multiple slip systems have to move simultaneously to satisfy the harmony of deformation near the grain boundaries, which causes dislocations to be blocked and makes moving across grain boundaries difficult, thus leading to strength enhancement. The grain is refined and the size of the grain boundary increases. The grain boundary sliding would deform with difficulty which results in a higher yield limit and a better wear-resistance property. According to the Hall–Petch equation [31,32,33,34], the relation between the yield limit and grain size can be described as

σ_s_ = σ_y_ + *Kd*^−1/2^ (3)

where σ_s_ is the yield limit, σ_y_ is the yield limit of a single crystal, *K* is the response factor of the grain boundary to deformation, and *d* is grain size. As *d* decreases, σ_s_ will increase, thus leading to a better wear-resistance property.

Furthermore, as the abrasion of the composite coating continues, the embedded nanodiamond particles will be exposed and inhibit the extension and exfoliation of the adhesion area due to their load-bearing role, which improves the adherence resistance of the coating. The nanodiamond particles also play an important role as lubricating agents, helping to weaken the abrasion.

As the dispersion/dislocation strengthening effect, grain refinement strengthening effect, and load-bearing and lubricating roles are all strongly related to the nanodiamond concentration in the composite coatings, an effective strategy to enhance the wear-resistance property should be increasing the number of embedded nanodiamond particles. However, a high concentration of nanodiamond particles will cause aggregation and thus results in performance loss.

In conclusion, the wear-resistance property of the nanodiamond composite coating appears to be superior to that of pure nickel film as a consequence of the synergic effect of all the physical characteristics mentioned above. The friction factors of all the composite coatings deposited here were smaller than pure nickel film and generally increased with the embedded nanodiamond concentration. When the concentration of nanodiamond particles in the plating solution was 8 g/L, the composite coating had the lowest friction factor and best wear-resistance performance.

The co-deposition of particles was mainly achieved through the two steps of weak adsorption and strong adsorption prior to being embedded into the coating [35]. Without considering the effects of the current density and other parameters, the greater the number of particles of nanodiamond particles present in the plating solution, the more weakly adsorbed the grains were by agitation and convection in the electroplating process, and, consequently, the greater the number of grains that were embedded into the composite coating. When the nanodiamond concentration in the solution exceeded 8.0 g/L, the nanodiamond content decreased due to three main reasons. First, when the nanodiamond content in the solution was quite high, the grains would be shielded from each other, far from the cathode surface and suspended in the solution, and elastic collisions between the grains would occur. Due to the direction of movement of the grain points to the cathode, a portion of the grains would be back-reflected between the cathode and the plating solution interface and, as a result, the number of grains incorporated into the composite coating would be reduced. Second, when very high nanodiamond grains in the solution exist, due to the limited surfactant and poor wettability, the agglomeration phenomenon would be aggravated, the sedimentation would be severe, the grain size would increase, the transmission speed in the solution would decrease, and the nanodiamond content in the composite coating would be reduced. Third, sufficient time was required to embed the nanodiamond particles in the plating solution into the composite coating as the adsorptive capacity was controlled by the corresponding nanodiamond particles and nickel ion transfer rate. Therefore, the nanodiamond content in the composite coating would not increase due to the unchanged capture ability of nickel, even though the number of nanodiamond particles transferred to the cathode increased.

When the current density increased, the diamond content in the composite coating increased at first and then decreased. The reasons for this were as follows: when the current density was lower, the convective phenomena caused by particle settling and agitation in the solution would inhibit the adsorption of nanodiamond particles onto the coating. Also, when the nanodiamond particles were weakly adsorbed on the cathode, it would require a long time to completely embed them into the nickel matrix to produce strong adsorption, and the migration rate of the nickel ions in the electric field action was slower. Therefore, the diamond content in the composite coating was reduced. When the current density was increased to 3.0 A/dm^2^, the transfer rate of the nickel matrix became faster; the nanodiamond particles were completely embedded into the nickel surface at a faster speed, which reduced the probability of shedding caused by sedimentation and played a certain role in the corresponding formation of the composite coating [18]. Furthermore, because the transmission of the nickel matrix was mainly controlled by electromigration, the current density continued to increase and the transmission rate continued to increase. For the nanodiamond particles with poor conductivity, the transmission in the plating solution mainly relied on convection caused by stirring and was irrelevant to the current density. Therefore, the deposition rate of the nickel matrix was relatively higher than the deposition rate of the captured nanodiamond particles. Generally, the content of nanodiamond particles in the composite coating was reduced. The nanodiamond particles were nonconductive; when they were embedded into the nickel matrix, the corresponding conductive area was lower, which indirectly increased the current density in the plating solution to increase the cathode polarization and finally decrease the nanodiamond content in the composite coating [36].

The effect of current density on the hardness of the composite coating was consistent with the corresponding effect of current density on the diamond content in the composite coating, representing the directly proportional relationship between the hardness of the composite coating and the diamond content: As the current density increased, the grain size of the composite coating varied and consequently, affected the hardness of the composite coating [37].

**Figure 13:**
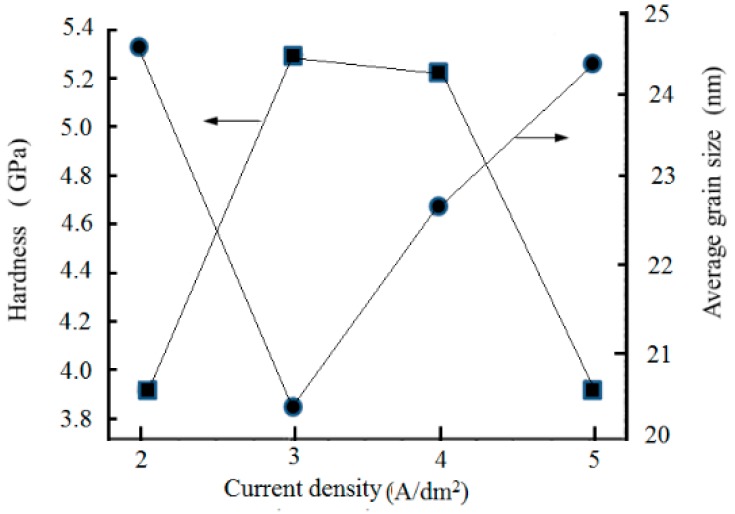
Relationship curve between current density and the grain size and hardness of the composite coating.

Figure 13 presents the variation of the grain size and hardness of the Ni/nanodiamond composite coating at various current densities. When the nanodiamond concentration in the plating solution was 8.0 g/L and the current density was 3.0 A/dm^2^, the hardness of the composite coating reached the highest value of 5.302 GPa and the average grain size was 20.4 nm.

### 3.5. Effect of Nanodiamond Concentration in the Plating Solution and Current Density on the Residual Stress of the Composite Coating

In this paper, the residual stress of composite coatings is measured by the X-ray diffraction method with the basic principle that the intensity of residual stress will bear on the spacing among coated crystal planes after the residual stress is produced by the metal materials. The residual stress is calculated with the following formula:

(4) $$\sigma =-\frac{E}{2\left(1+\nu \right)}\cot\theta_{0}\frac{\partial 2\theta }{\partial \sin^{2}\psi }$$

where *E* and ν represent the elastic modulus and Poisson’s ratio of the coatings, respectively. The standard diffraction angle of the coatings is found to be θ_0_ = 121.936° from the X-ray diffraction manual. The corresponding diffraction angle of the measured crystal surface is 2θ. ψ is the angle between the coatings’ normal line and the diffraction crystal surface normal line, being 0°, 15°, 25°, 35° and 45°, respectively. X-rays can penetrate the surface layer around 30 μm. The thickness of the nanodiamond–nickel composite coating is around 20 μm, conforming to the test conditions.

**Figure 14:**
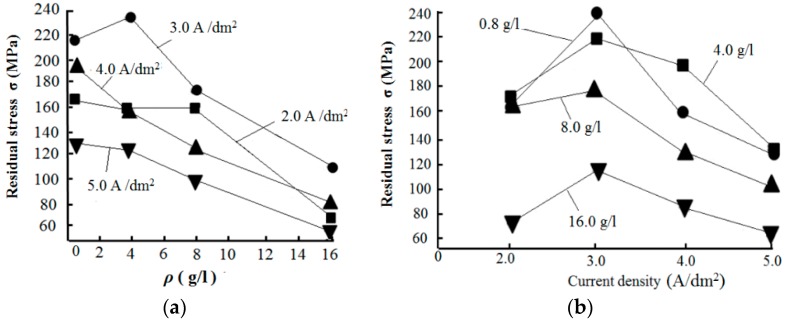
Effect of (**a**) nanodiamond concentration in the plating solution and (**b**) current density on the residual stress of the composite coating.

Figure 14 presents the effect of the nanodiamond concentration in the plating solution and current density on the residual stress of the Ni/nanodiamond composite coating.

Figure 14a demonstrates that the residual stress of the Ni/nanodiamond composite coating was tensile, regardless of the nanodiamond concentration. When the nanodiamond concentration in the plating solution was 0.8 g/L, the residual stress of the composite coating reached a maximum of 238.37 MPa. The residual stress of the composite coating gradually decreased as the nanodiamond concentration increased because the preferential orientation of the crystal face (200) in the composite coating was enhanced, and the decrease of residual stress was related to the (200) texture coefficient variation. When the nanodiamond concentration increased from 8.0 to 16.0 g/L, the nanodiamond content in the composite coating decreased, subsequently decreasing the lattice distortion and residual stress, consistent with the conclusion given in [38].

Figure 14b shows that the residual stress of the composite coating ranged from 80 to 240 MPa and it was also tensile. When the current density increased from 2.0 to 5.0 A/dm^2^, the residual stress of the composite coating increased at first and then decreased. Sajjadnejad et al. reported that the presence of the nanodiamond (ND) particles in the nickel substrate led to increased residual stress [20].

Due to the change in the current density, the nucleation and growth in the electrodeposition also changed, affecting the residual stress of the composite coating [39,40]. As the current density increased, the polarization increased, and the crystal nucleation rate became faster, resulting in a decreasing grain size. The numbers of vacancies, grain boundaries, dislocations, and other defects in the composite coatings increased, and the hydrogen evolution reaction was also enhanced. Therefore, the residual stress in the composite coating decreased with the increasing current density in the electrodeposition to a certain extent. When the current density increased to 5.0 A/dm^2^, due to the lack of nickel ions near the cathode, the hydrogen evolution reaction was intensified, the compactness of the composite coating was reduced, and the stress was released through the gap between the particles, resulting in a lower residual stress of the composite coating.

### 3.6. Performance Comparison between the Nickel Coating and Ni/Nanodiamond Composite Coating

With a current density at 3.0 A/dm^2^, a temperature of 45 °C, and a pH value of 3.5 [21], the surface morphology, crystal phase structure, and the mechanical properties of the two coatings were compared when the nanodiamond concentrations in the plating solution were 0 and 8.0 g/L, respectively.

**Figure 15:**
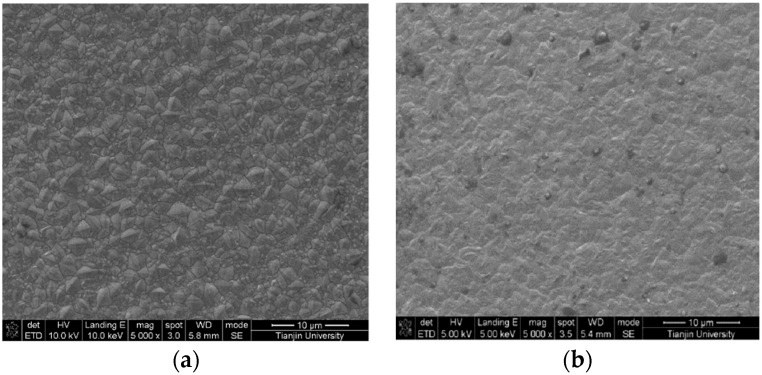
SEM images of the Ni coating (**a**) and Ni/nanodiamond composite coating (**b**).

Figure 15 presents the surface morphology of the Ni and Ni/nanodiamond composite coating. The grains on the surface of the nickel coating were in a pyramidal polyhedron shape, and the grain size and distribution were relatively uniform. However, more grain boundaries and higher-sized gaps existed between the grains. In contrast, the compactness among the grains in the composite coating was apparent. The nanodiamond particles on the surface were completely embedded into the nickel matrix, and the distribution was relatively uniform. The lattice distortion increased and the gap between the grains could be supplemented, exerting a strengthening effect.

**Figure 16:**
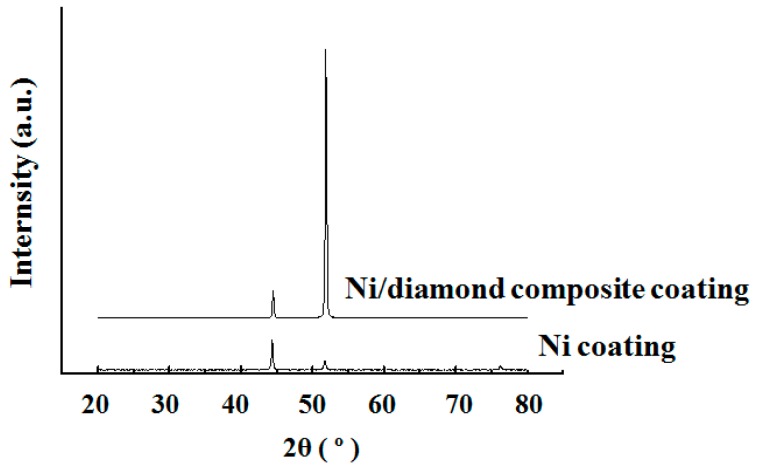
XRD spectra of the Ni/nanodiamond composite and Ni coatings.

Figure 16 presents the XRD patterns of the Ni/nanodiamond composite and Ni coatings. The nickel coating showed preferential orientation along the (111) crystal face, the diffraction peak was sharper, and the corresponding grain size was 30.8 nm [21], whereas the composite coating showed preferential orientation along the (200) crystal face, the diffraction peaks of (111) and (200) crystal faces were significantly broadened, and the grain size was reduced to 20.4 nm. Therefore, it could be concluded that the deposition of nanodiamond particles onto the matrix surface formed a higher number of nucleation points for the deposition of nickel atoms, allowing the nickel atoms to complete the nucleation and deposition processes quite rapidly with uniform distribution. Furthermore, as the nanodiamond particles were deposited onto the surface of the matrix, the real deposition area was reduced, the true current density of the electrodeposition increased, and the cathodic overpotential increased, which had a certain effect on the crystal phase structure of the coating.

The hardness of the composite coating reached a value of 5.302 GPa, which was greatly improved compared to the nickel coating of 3.8 GPa. The elastic modulus of the composite coating reached 254.356 GPa, which was higher than the value for the nickel coating of 238 GPa because the elastic modulus of the nanodiamond particles was higher (1141 GPa).

The residual stress of the composite coating was higher than the nickel coating stress to a certain extent because the nanodiamond particles in the composite coating have a greater effect on the lattice distortion. Accordingly, the defects of the composite coating, including the dislocations and vacancies, also increased, the grain size became lower, and consequently the grain boundary increased, which inhibited the dislocation movement in the composite coating. Therefore, the Ni/nanodiamond composite coating had a higher hardness and elastic modulus.

## 4. Conclusions

The nanodiamond concentration in the plating solution and current density of the cathode had corresponding effects on the surface morphology, texture, hardness, residual stress, and friction properties of the Ni/nanodiamond composite coating. The preferential orientation of the composite coating varied from the (111) crystal orientation of the pure nickel coating to the (200) crystal orientation of the composite coating. When the nanodiamond concentration in the plating solution was 8.0 g/L at a current density of 3.0 A/dm^2^, the electroplated Ni/nanodiamond composite coating had the best tribological performance, a friction factor that was maintained at around 0.1, and hardness up to 5.302 GPa. The average grain size of the Ni/nanodiamond composite coatings obtained under similar conditions was reduced to 20.4 nm, with preferential orientation observed along the (200) crystal face.

## Figures and Tables

**Table 1:** Composition of degreasing solution and process parameters.

Compositions and Parameter	Chemical De-Oiling	Electrochemical De-Oiling
NaOH	65 g/L	15 g/L
Na_2_CO_3_	17.5 g/L	55 g/L
Na_3_PO_4_·12H_2_O	17.5 g/L	35 g/L
Na_2_SiO_3_·9H_2_O	5 g/L	7.5 g/L
Temperature	70 °C	70 °C
Time	2 min	1 min
Electricity	–	0.2 A

**Table 2:** Contents of impurity elements in ultra-dispersed nanodiamond powder (% by mass).

**Element**	**Fe**	**Cr**	**Si**	**Al**	**Na**	**K**	**Cu**
Content	0.150	0.070	0.300	0.005	0.030	0.002	0.005
**Element**	**Ca**	**Mg**	**Mn**	**Ti**	**Pb**	**Incombustible substance**	**Total**
Content	0.002	0.005	0.001	0.010	0.001	0.95	1.531

## References

[1] Low C.T.J., Wills R.G.A., Walsh F.C. (2006). Electrodeposition of composite coatings containing nanoparticles in a metal deposit. Surf. Coat. Technol..

[2] Vaezi M.R., Sadrnezhaad S.K., Nikzad L. (2008). Electrodeposition of Ni–SiC nanocomposite coatings and evaluation of wear and corrosion resistance and electroplating characteristics. Colloid Surf. A Physicochem. Eng. Asp..

[3] Shi L., Sun C., Gao P., Zhou F., Liu W. (2006). Mechanical properties and wear and corrosion resistance of electrodeposited Ni–Co/SiC nanocomposite coating. Appl. Surf. Sci..

[4] Lee H.K., Lee H.Y., Jeon J.M. (2007). Codeposition of micro- and nano-sized SiC particles in the nickel matrix composite coatings obtained by electroplating. Surf. Coat. Technol..

[5] Srivastava M., Grips V.K.W., Rajam K.S. (2007). Electrochemical deposition and tribological behaviour of Ni and Ni–Co metal matrix composites with SiC nanoparticles. Appl. Surf. Sci..

[6] Lampke T., Wielage B., Dietrich D., Leopold A. (2006). Details of crystalline growth in co-deposited electroplated nickel films with hard (nano) particles. Appl. Surf. Sci..

[7] Yao Y., Yao S., Zhang L., Wang H. (2007). Electrodeposition and mechanical and corrosion resistance properties of Ni–W/SiC nanocomposite coatings. Mater. Lett..

[8] Gyftou P., Pavlatou E.A., Spyrellis N. (2008). Effect of pulse electrodeposition parameters on the properties of Ni/nano-SiC composites. Appl. Surf. Sci..

[9] Surender M., Basu B., Balasubramaniam R. (2004). Wear characterization of electrodeposited Ni–WC composite coatings. Tribol. Int..

[10] Feng Q.Y., Li T.J., Yue H.Y., Qi K., Bai F.D., Jin J.Z. (2008). Preparation and characterization of nickel nano-Al_2_O_3_ composite coatings by sediment co-deposition. Appl. Surf. Sci..

[11] Kuo S.L., Chenb Y.C., Ger M.D., Wu W.H. (2004). Nano-particles dispersion effect on Ni/Al_2_O_3_ composite coatings. Mater. Phys. Chem..

[12] Wang L.-P., Gao Y., Xue Q.-J., Liu H.-W., Xu T. (2004). Effect of nano-diamond particulates on the microstructure and wear-resistance of electrodeposited Ni-matrix coatings. Tribology.

[13] Petrov I., Detkov P., Drovosekov A., Ivanov M.V., Tyler T., Shenderova O., Voznecova N.P., Toporov Y.P., Schulz D. (2006). Nickel galvanic coatings co-deposited with frantions of detonation nanodiamond. Diam. Relat. Mater..

[14] Ogihara H., Safuan M., Saji T. (2012). Effect of electrodeposition conditions on hardness of Ni–B/diamond composite films. Surf. Coat. Technol..

[15] Shrestha N.K., Takebe T., Saji T. (2006). Effect of particle size on the co-deposition of diamond with nickel in presence of a redox-active surfactant and mechanical property of the coatings. Diam. Relat. Mater..

[16] Abdoli M., Rouhaghdam A.S. (2013). Preparation and characterization of Ni–P/nanodiamond coatings: Effects of surfactants. Diam. Relat. Mater..

[17] Wang L., Gao Y., Liu H., Xue Q., Xu T. (2005). Effects of bivalent Co ion on the co-deposition of nickel and nano-diamond particles. Surf. Coat. Technol..

[18] Lee W.H., Tang S.C., Chung K.C. (1999). Effects of direct current and pulse-plating on the co-deposition of nickel and nanometer diamond powder. Surf. Coat. Technol..

[19] Huang W., Zhao Y.W., Wang X.L. (2013). Preparing a high-particle-content Ni/diamond composite coating with strong abrasive ability. Surf. Coat. Technol..

[20] Sajjadnejad M., Omidvar H., Javanbakht M., Mozafari A. (2017). Textural and structural evolution of pulse electrodeposited Ni/diamond nanocomposite coatings. J. Alloy. Compd..

[21] Zhao Y., Liu M., Feng L., Meng Y., Li F., Chen Z. (2014). Optimization of technology for electrodeposition of nickel coating on Q235A steel substrate. Mater. Prot..

[22] Volkov D.S., Proskurnin M.A., Korobov M.V. (2014). Elemental analysis of nanodiamonds by inductively-coupled plasma atomic emission spectroscopy. Carbon.

[23] Lv B., Hu Z., Wang X., Xu B. (2013). Effect of current density on the microstructure and properties of plated nickel coating. China Surf. Eng..

[24] Wang Y., Yang C., He J., Wang W., Mitsuzak N., Chen Z. (2016). Effects of choline chloride on electrodeposited Ni coating from a Watts-type bath. Appl. Surf. Sci..

[25] Ma C., Wang S.C., Wang L.P., Walsh F.C., Wood R.J.K. (2013). The electrodeposition and characterization of low-friction and wear-resistant Co–Ni–P coatings. Surf. Coat. Technol..

[26] Han F., Li S., Zhu L., Nie Y., Yu K., Wang J., Su T., Hu M., Xiao H. (2018). Application of laser raman spectroscopy method in research of diamond. J. Synth. Cryst..

[27] Catledge S.A., Vohra Y.K. (1996). Micro-Raman stress investigations and X-ray diffraction analysis of polycrystalline diamond (PCD) tools. Diam. Relat. Mater..

[28] Fabisiak K., Banaszak A., Kaczmarski M., Kozanecki M. (2006). Structural characterization of CVD diamond films using Raman and ESR spectroscopy methods. Opt. Mater..

[29] Molina J.M., Saravanan R.A., Narciso J., Louis E. (2004). Surface modification of 2014 aluminium alloy—Al_2_O_3_ particles composites by nickel electrochemical deposition. Mater. Sci. Eng. A.

[30] Arpón R., Molina J.M., Saravanan R.A., García-Cordovilla C., Louis E., Narciso J. (2003). Thermal expansion behaviour of aluminium/SiC composites with bimodal particle distributions. Acta Mater..

[31] Hetong G. (2007). The Technique of Composite Electroplating.

[32] Gül H., Kilic F., Uysal M. (2012). Effect of particle concentration on the structure and tribological properties of submicron particle SiC reinforced Ni metal matrix composite (MMC) coatings produced by electrodeposition. Appl. Surf. Sci..

[33] Wang M., Jiang B., Xu B., Ma S., Dong S. (2005). Microstructure and fretting wear behavior of Ni based composite coatings reinforced by SiO_2_ nanoparticles. Tribology.

[34] Xue Y., Zhu D., Jin G., Zhao F. (2005). Friction and wear properties of electrodeposited Ni–La_2_O_3_ nanocomposite coatings. Tribology.

[35] Guglielmi N. (1972). Kinetics of deposition of inert particles from electrolytic baths. J. Electrochem. Soc..

[36] Pushpavanam M., Manikandan H., Ramanathan K. (2007). Preparation and characterization of nickel-cobalt-diamond electro-composites by sediment co-deposition. Surf. Coat. Technol..

[37] Sohrabi A., Dolati A., Ghorbani M. (2010). Nanomechanical properties of functionally graded composite coatings: Electrodeposited nickel dispersions containing silicon micro- and nanoparticles. Mater. Chem. Phys..

[38] AriGur P., Sariel J., Vemuganti S. (2007). Residual stresses and texture in Ni/SiC nanocomposite coating. J. Alloy. Compd..

[39] Pathak S., Guinard M. (2011). Influence of lower current densities on the residual stress and structure of thick nickel electrodeposits. Surf. Coat. Technol..

[40] Mizushima I., Tang P.T., Hansen H.N. (2006). Residual stress in Ni–W electrodeposits. Electrochim. Acta.

